# Supplementary material for: Designed miniproteins potently inhibit and protect against MERS-CoV
Source: bioRxiv. 2024 Nov 4:2024.11.03.621760. Preprint. [Version 1] doi: 10.1101/2024.11.03.621760 (PMC11580849; doi:10.1101/2024.11.03.621760)
Supplement: Supplement 1 [file NIHPP2024.11.03.621760v1-supplement-1.pdf]

bioRxiv preprint doi: https://doi.org/10.1101/2024.11.03.621760; this version posted November 4, 2024. The copyright holder for this preprint (which was not certified by peer review) is the author/funder, who has granted bioRxiv a license to display the preprint in perpetuity. It is made available under aCC-BY 4.0 International license.

Supplementary Tables

Table S1. X-ray crystallography data collection and refinement statistics of the cb3-MERS-CoV RBD complex.

	MERS-CoV RBD:cb3	HALC3_919
PDB ID	9DGO	9C7Z
Space Group	$P2_12_12_1$	P 21 3
**Cell dimensions**		
a, b, c (Å)	61.619, 76.291, 136.235	78.373 78.373 78.373
α, β, γ (°)	90, 90, 90	90, 90, 90
Resolution range (Å)	31.55-1.85 (1.95-1.85)	55.42-2.1 (2.16-2.1)
CC1/2	0.999 (0.79)	0.999 (0.681)
Rmerge	0.023 (0.378)	0.059 (0.988)
I/σ(I)	14.7 (1.9)	15.5 (2.1)
Completeness (%)	100 (99.9)	100 (99.9)
Redundancy	2 (2)	8.8 (7.9)
**Refinement**		
No. reflections	55567	84558
Rwork/Rfree	18.96/22.06	23.33/23.85
Nº of atoms		
Protein	4116	988
Ligand		8
Water	307	23
B factor (Wilson plot)	33.05	48.68
**R.m.s. deviations**		
Bond lengths (Å)	0.007	0.009
Bond angles (°)	0.849	1.11
Ramachandran favored (%)	99.43	100
Ramachandran allowed (%)	0.57	0.0
Ramachandran outliers (%)	0.0	0.0

- Data in parentheses are for the highest resolution shell
- $R_{merge} = \sum(\sum|I_i - \langle I \rangle|/\sum|I|)$, where the first ∑ is the sum over all reflections, and the second ∑ is the sum over all measurements of a given reflection, with I_i being the *i*th measurement of the intensity of the reflection and $\langle I \rangle$ the average intensity of that reflection.

bioRxiv preprint doi: https://doi.org/10.1101/2024.11.03.621760; this version posted November 4, 2024. The copyright holder for this preprint (which was not certified by peer review) is the author/funder, who has granted bioRxiv a license to display the preprint in perpetuity. It is made available under aCC-BY 4.0 International license.

- $R_{work}/R_{free} = \sum(|F_o| - \langle|F_c|\rangle)/\sum|F_o|$, where $\langle|F_c|\rangle$ is the expectation of $|F_c|$ under the probability model used to define the likelihood function. The sum is overall reflections.

bioRxiv preprint doi: https://doi.org/10.1101/2024.11.03.621760; this version posted November 4, 2024. The copyright holder for this preprint (which was not certified by peer review) is the author/funder, who has granted bioRxiv a license to display the preprint in perpetuity. It is made available under aCC-BY 4.0 International license.

Table S2. S glycoprotein mutations in MERS-CoV S variants used in this study relative to MERS-CoV S EMC/2012 (NC_019843.3). The mutations located at the receptor binding motif are highlighted in red.

MERS-CoV Variant	Genbank Accession Numb	Spike Protein Mutations
United Kingdom/H123990006/2012	NC_038294.1	L506F, Q1020H
2cJordan-N3/2012	AHY21469.1	G94V, H194Y, L301R, I879T, A1158S
Korea/Seoul/168-1-2015	KT374056.1	H91Y, D510G, Q1020R
Camel/Kenya/M23C14/2019	OK094446.1	V26A, D158Y, H194Y, S390F, L450F, V R626P, A1158S

Table S3. Summary of binding kinetics for monomeric and trimeric miniproteins. K_Ds were determined through global langmuir 1:1 model fitting.

Design	k_{on} ($M^{-1}s^{-1}$)	k_{off} (s^{-1})	K_D (M)
cb3	3.0E6	1.1E-2	3.7E-9
cb4	3.5E5	2.2E-2	6.1E-8
cb6	4.2E5	1.8E-2	4.4E-8
nHALC3_919_cb3	5.6E5*	6.0E-5*	1.1E-10*
nHALC3_104_cb3	6.5E5*	5.3E-5*	8.1E-11*
nHALC3_110_cb3	4.8E5*	4.7E-5*	9.7E-11*
cHALC3_110_cb3	4.8E4*	2.6E-3*	5.4E-8*
nSB175	6.8E5*	4.2E-5*	6.2E-11*

* indicate apparent affinity values due to avidity of binding of the trimeric miniproteins to the prefusion MERS-CoV S trimer

bioRxiv preprint doi: https://doi.org/10.1101/2024.11.03.621760; this version posted November 4, 2024. The copyright holder for this preprint (which was not certified by peer review) is the author/funder, who has granted bioRxiv a license to display the preprint in perpetuity. It is made available under aCC-BY 4.0 International license.

Table S4. Designed sequences of miniproteins and homo-oligomer domains described in the text. Each trimer was tested as an N- and C-terminal fusion with respect to the miniprotein cb3. All constructs were expressed with MSG - design - GS - SNAC tag - 6x his, as described in methods.

Design	Sequence
cb3	SPVKRFVREVLEEAEEAYEKGDRRQFEELLWLAEWAARDANDEELEEEIREFEKEVK
cb4	SPVKRFIREVLEEAEEAYEGGDRHQFEELLWLANWAARDANDEEAEEEIREFEKEVK
cb6	SGAKRFVREVLEEAEEAYEKGDRRQFEELLWLAQWAARDANDEELEEEIREFEKEVK
SB175	SEALEELEKALRELKKSTDELERSTEELEKNPSEDALVENNRLIVENNKIIVEVLRIIAKVLK
HALC3_104	KRIDEIESKLKHLEEFTTHLIKLMETMLELLKLVSDGKSDSEEYKELLEKAEEYLKQATEAAKKI
HALC3_110	LEQILEELTELLERVDEIPLREALKRMLELLVRVTQELKEVKDKVESLEKHLEELDKRVEEIEKK
HALC3_114	VDEKEVKERFEEIESRLEELESKVREVEKKVEEVKKESDEKIDQLKTEFETKYNQINNEINTLKN
HALC3_118	MTRLEQLLAQGVDPFEVLREKIEKLKEIWKKYEEAKGEEKERYRDELLKLMMEVLELMVELLSRR
HALC3_919	SEELLEELRELLERLQELLELIEQGKITPEQLREAIALLIEVLQILYEALRELAEQLQRLREELG

Table S5. Sequences of the different linkers used between the trimerization domain nSB175 and the miniprotein cb3. The "n" refers to the position of the miniprotein cb3 relative to the trimerization domain. All constructs were expressed with MSG preceding the designed sequence and SNAC tag - 6x his at the C terminus, as described in methods.

Design	Sequence
nSB175_cb3	cb3-GSG-SB175
Linker 1	cb3-GS-SB175
Linker 2	cb3-GSGS-SB175
Linker 3	cb3-GSGSGS-SB175
Linker 4	cb3-GSGSGSGS-SB175
Linker 5	cb3-GSGSGSGSGS-SB175
Linker 6	cb3-GGGS-SB175
Linker 7	cb3-GGGSGGGS-SB175
Linker 8	cb3-GGGSGGGSGGGS-SB175

bioRxiv preprint doi: https://doi.org/10.1101/2024.11.03.621760; this version posted November 4, 2024. The copyright holder for this preprint (which was not certified by peer review) is the author/funder, who has granted bioRxiv a license to display the preprint in perpetuity. It is made available under aCC-BY 4.0 International license.

Table S6. List of IC_{50} values obtained from the neutralization curves shown in Fig S3 expressed in nanomolar. Monomeric miniprotein cb3 was used as a reference to highlight the improved neutralization exhibited by the trimerization of cb3. Monomeric miniprotein cb4 was used as a negative control. The two IC_{50} values for each pseudotyped virus correspond to two distinct biological experiments performed with two batches of pseudovirus and one batch or miniprotein. The “n” and “c” indices refer to the position N- or C-terminus of the miniprotein cb3 relative to the indicated trimerization domain. NN: No Neutralization. Limit of detection (LOD) of miniproteins is between $5x10^2$-10^3 nM (see Fig S3).

IC50s (nM)						
**miniprotein**	**EMC/2012**		**Jordan/2012**		**United Kingdom/2012**	
nSB175_cb3	5.1	1.6	5.9	3	2.6	2.8
nHALC3_104	7.2	1.45	4	1.8	2.6	1.9
nHALC3_118	7.2	1.4	4	2.7	1.5	1.6
nHALC3_919	5.9	1.7	10.3	5.7	5.4	4.9
cSB175_cb3	9.4	2.4	14	3.9	1	NN
cHALC3_104	1.7	1.7	23.3	9.2	26.8	32.9
cHALC3_110	14.8	22.9	18.8	23.2	LOD	LOD
cHALC3_114	0.4	1	8.9	3.1	LOD	LOD
cHALC3_118	41	12.7	26.3	17.8	LOD	LOD
cHALC3_919	15.3	5	17.8	19.7	LOD	LOD
cb3	200.3	115.8	167.6	169	607.2	355.3
cb4	LOD	LOD	LOD	LOD	LOD	LOD

Table S7. IC_{50} values obtained from the experiments shown in Fig S4 are expressed in nanomolar. Monomeric miniprotein cb3 was used as a reference and monomeric miniprotein cb4 was used as a negative control. The two IC_{50} values correspond to two different biological replicates using two batches of pseudovirus and one batch or miniprotein. The “n” and “c” indices refer to the position N- or C-terminus of miniprotein cb3 relative to the indicated trimerization domain. Limit of detection (LOD) for cb4 and cHALC3_110 is 10^3 nM (see Fig S4).

IC50s (nM)										
**miniprotein**	**EMC/2012**		**Jordan/2012**		**United Kingdom/2012**		**Seoul/2015**		**Kenya/2019**	
nSB175_cb3	3.4	2.8	2.9	2.5	4.9	4.7	2.2	1	3.9	1
nHALC3_104	2.4	2	1.7	1.9	4	4.5	0.6	1.3	1.7	0.45
nHALC3_118	4	3.2	2.8	3.7	5.7	6.8	2.3	2.1	3.2	1.7
cHALC3_110	10.9	6.4	8.9	6.8	LOD	LOD	17.8	21.7	4.8	2.3
cb3	109.5	229	114.4	146.7	703.9	642	252.2	212.6	117.9	97.17
cb4	LOD	LOD	LOD	LOD	LOD	LOD	LOD	LOD	LOD	LOD

bioRxiv preprint doi: https://doi.org/10.1101/2024.11.03.621760; this version posted November 4, 2024. The copyright holder for this preprint (which was not certified by peer review) is the author/funder, who has granted bioRxiv a license to display the preprint in perpetuity. It is made available under aCC-BY 4.0 International license.

Table S8. CryoEM data collection and refinement statistics.

**Data collection and processing**	**MERS-CoV S in complex with cb3-GSG-SB175, linker 1 (Global refinement, 2 RBDs engaged)**	**MERS-CoV S in complex with cb3-GSG-SB175, linker 1 (Global refinement, 3 RBDs engaged)**	**MERS-CoV S in complex with cb3-GGGSGGGS-SSB175, linker 7 (Global refinement)**	**MERS-CoV S in complex with cb3-GGGSGGGS-SSB175, linker 7 (Local refinement)**	**MERS-CoV S in complex with cb3-GGGSGGGS-SB175, linker 7 (Global refinement after focused classification, 3 RBDs engaged)**
Magnification	105,000	105,000	105,000	105,000	105,000
Voltage (kV)	300	300	300	300	300
Electron exposure (e^{-}/Å^2)	53.25	53.25	53.25	53.25	53.25
Defocus range (-μm)	0.8 - 1.7	0.8 - 1.7	0.8 - 1.7	0.8 - 1.7	0.8 - 1.7
Pixel size (Å)	0.829	0.829	0.829	0.829	0.829
Symmetry imposed	C1	C1	C1	C1	C1
Initial number of particles	615,255	615,255	986,712	986,712	986,712
Final number of particles	271.037	79.019	538.654	169.944	63.523
Map resolution (Å)	2.9	3.3	2.6	3.2	3.0
FSC threshold	0.143	0.143	0.143	0.143	0.143
Map sharpening B factor (Å^2)	-12.13	19.11	-46.60	-66.35	-21.03
**Validation**					
MolProbity score	N/A	N/A	N/A	1.25	N/A
Clash score	N/A	N/A	N/A	4.75	N/A
Poor rotamers (%)	N/A	N/A	N/A	0.00	N/A
**Ramachandran plot**					
Favored (%)	N/A	N/A	N/A	98.56	N/A
Allowed (%)	N/A	N/A	N/A	1.44	N/A
Disallowed (%)	N/A	N/A	N/A	0.0	N/A
**Entry codes**	EMD:46947	EMD:-46952	EMD:46955	PDB: 9DKK<br>EMDB: 46960	EMD:46957

bioRxiv preprint doi: https://doi.org/10.1101/2024.11.03.621760; this version posted November 4, 2024. The copyright holder for this preprint (which was not certified by peer review) is the author/funder, who has granted bioRxiv a license to display the preprint in perpetuity. It is made available under aCC-BY 4.0 International license.

Supplementary Figures

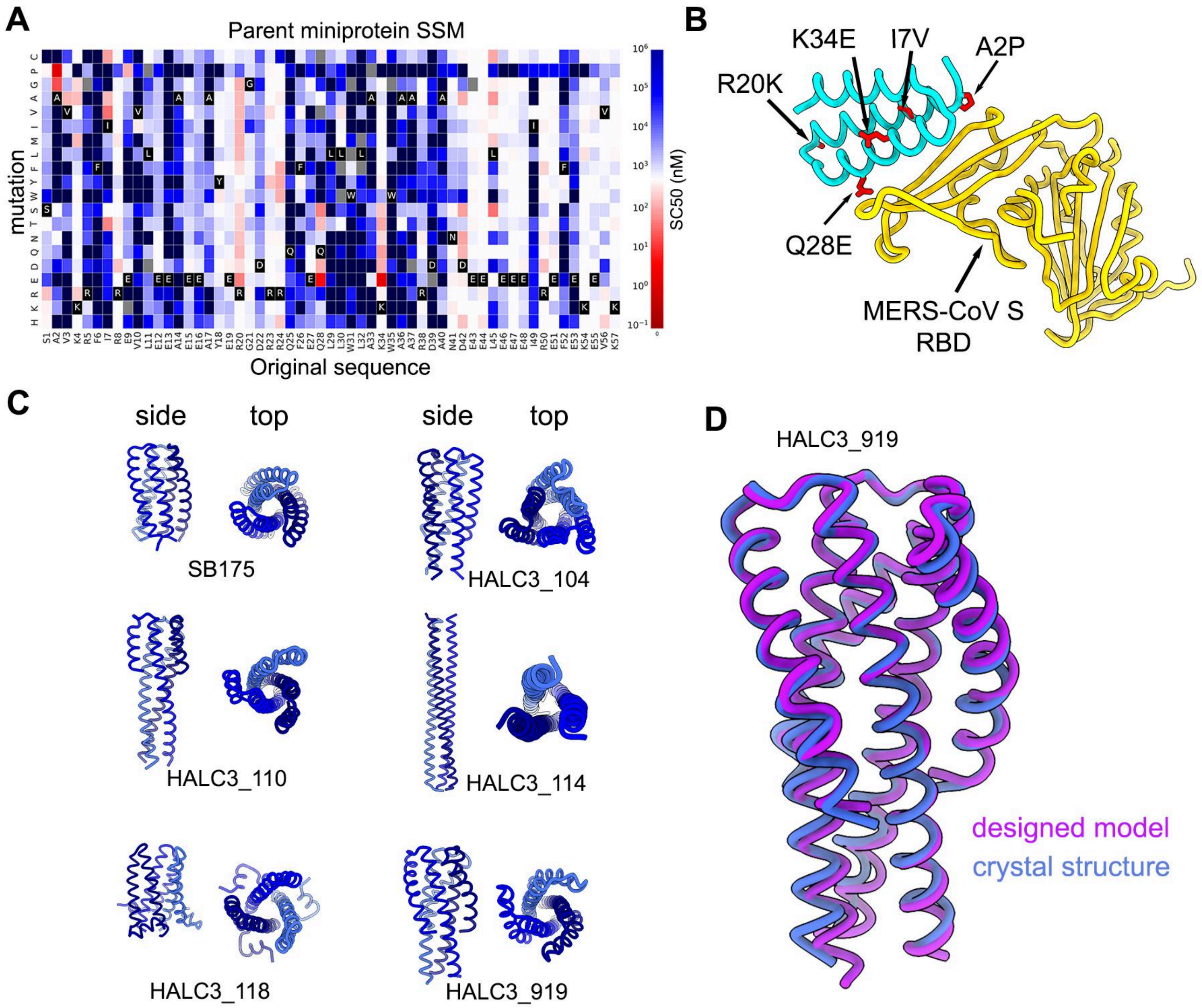

Fig S1. Optimization of MERS-CoV S RBD-directed miniproteins. A. Site saturation mutagenesis of the initial hit, designated parent miniprotein from which a combinatorial yeast surface display library was produced by selecting affinity enhancing point mutations. For each point mutation, the concentration that achieves 50 % of the saturating binding signal on yeast (SC50*(32)*) was calculated and plotted as enhancing affinity (red), reducing affinity (blue) or not affecting affinity (white) as compared to the SC50 of the parental design. The amino acid residue identity at each position of the parent

bioRxiv preprint doi: https://doi.org/10.1101/2024.11.03.621760; this version posted November 4, 2024. The copyright holder for this preprint (which was not certified by peer review) is the author/funder, who has granted bioRxiv a license to display the preprint in perpetuity. It is made available under aCC-BY 4.0 International license.

miniprotein is colored black with white text, with all possible amino acid substitutions for that position following the y-axis. Gray indicates mutations that were not present in the library. **B.** Selected mutations from the parent miniprotein to yield the cb3 design are indicated on the ribbon diagram of the complex X-ray structure. **C**. Models of trimerization motifs fused N- or C-terminally to the cb3 miniprotein. **D**. Crystal structure of the HALC3_919 trimerization domain superposed to the design model, yielding an r.m.s.d of 1.52 Å across all Cα in the homotrimer.

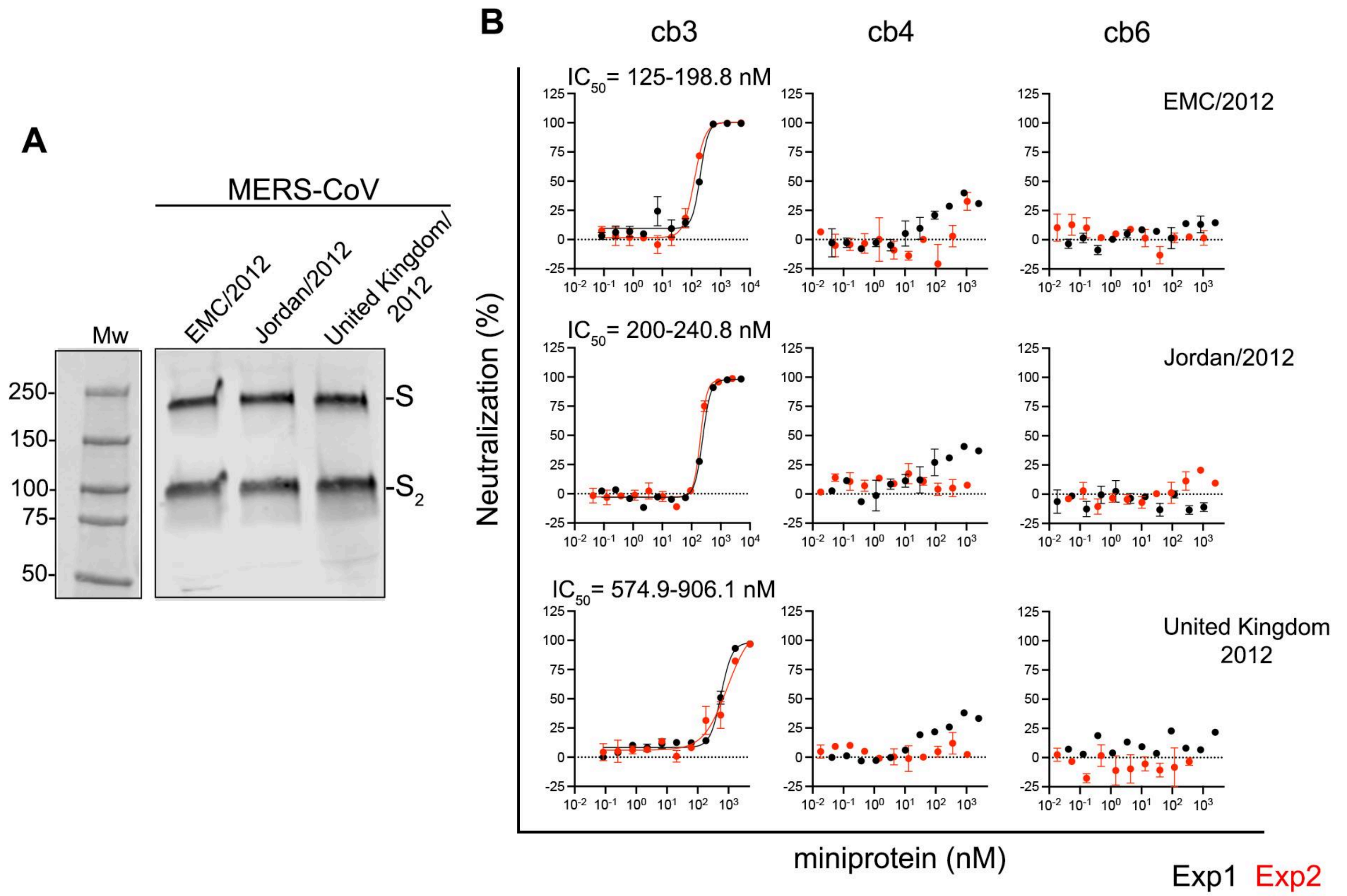

Fig S2. Inhibition of MERS-CoV S-mediated entry into VeroE6-TMPRSS2 cells by monomeric designed miniproteins. **A**, Western blot analysis of VSV pseudotyped particles harboring MERS-CoV EMC/2012, Jordan/2012 or United Kingdom/2012 S detected using the B6 stem-helix monoclonal antibody*(48)* as a primary antibody. Full-length S and S_2 subunit bands are indicated on the right-hand side of the blot. **B**, Concentration-dependent inhibition of MERS-CoV S pseudovirus entry into VeroE6-TMPRSS2 cells for MERS-CoV S EMC/2012, Jordan/2012 and United Kingdom/2012. Exp 1 and Exp 2 correspond to two biological experiments performed with different preparations of pseudotyped viruses and one preparation of miniprotein. Error bars represent the standard error of the mean (SEM) of the technical duplicates. Fits are shown only when neutralization was detected.

bioRxiv preprint doi: https://doi.org/10.1101/2024.11.03.621760; this version posted November 4, 2024. The copyright holder for this preprint (which was not certified by peer review) is the author/funder, who has granted bioRxiv a license to display the preprint in perpetuity. It is made available under aCC-BY 4.0 International license.

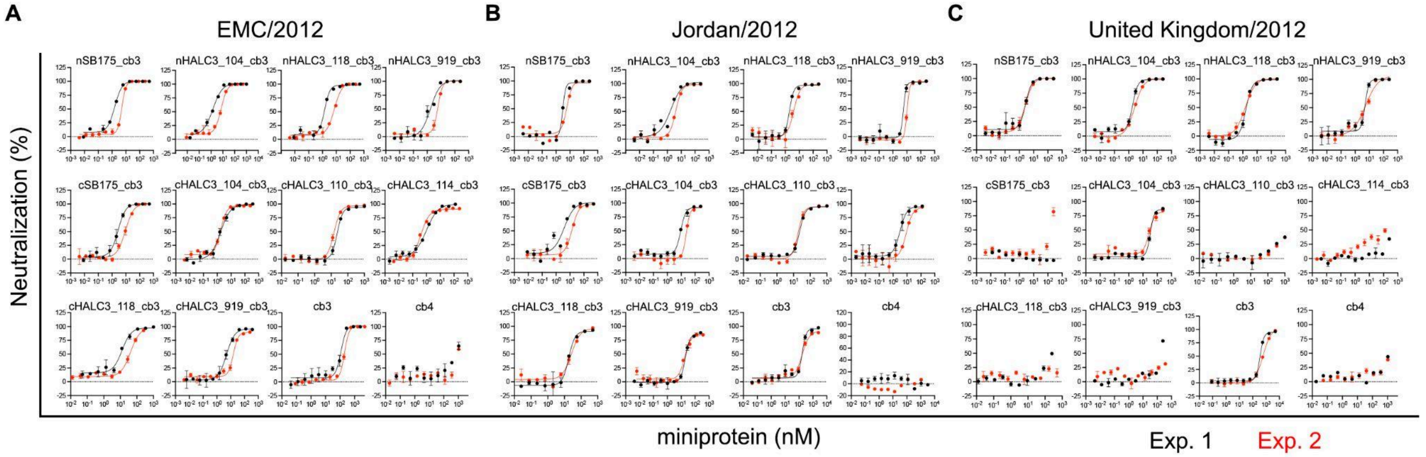

Fig S3. Inhibition of MERS-CoV S-mediated entry into VeroE6-TMPRSS2 cells by trimeric miniproteins. A-C, MERS-CoV EMC/2012 (**A**), Jordan/2012 (**B**) and United Kingdom/2012 (**C**) S VSV pseudovirus-mediated entry in the presence of various dilutions of the indicated miniproteins. Monomeric miniprotein cb3 was used as a reference and cb4 as negative control of neutralization. Exp 1 and Exp 2 correspond to two biological experiments performed with different preparations of pseudotyped viruses and one preparation of miniprotein. Error bars represent the standard error of the mean (SEM) of the technical duplicates. Fits are shown only when neutralization was detected.

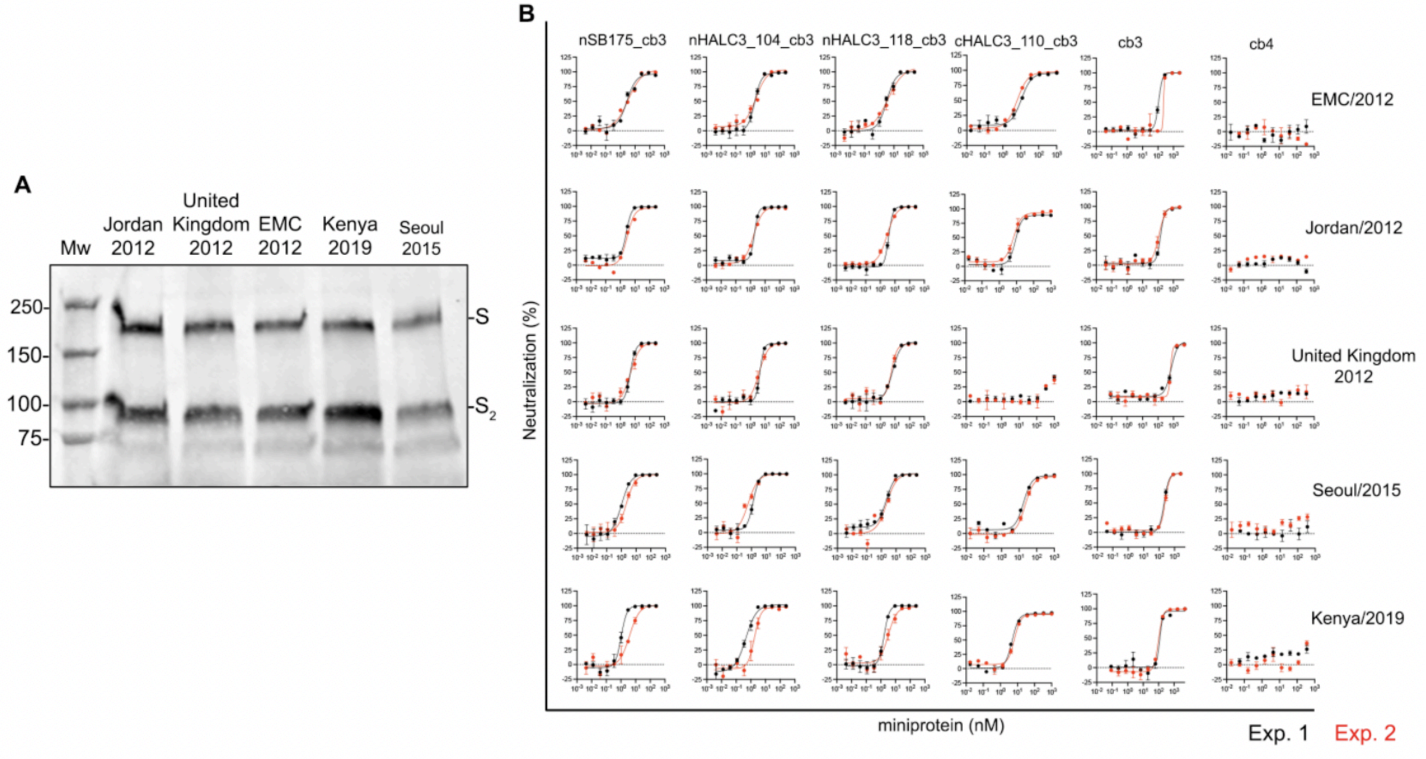

Fig S4. Inhibition of MERS-CoV S-mediated entry into VeroE6-TMPRSS2 cells by selected trimeric miniproteins. A. Western blot analysis of VSV pseudotyped particles harboring the indicated MERS-CoV S variants detected using the stem-helix monoclonal antibody B6*(48)* as a primary antibody Mw, molecular weight ladder. Full-length S and S_2 subunit bands are indicated on the right-hand side of the blot. **B.** MERS-CoV EMC2012, Jordan/2012, United Kingdom/2012, Kenya/2019 and Seoul/2015 S VSV pseudovirus entry in the presence of various dilutions of the indicated miniproteins.

bioRxiv preprint doi: https://doi.org/10.1101/2024.11.03.621760; this version posted November 4, 2024. The copyright holder for this preprint (which was not certified by peer review) is the author/funder, who has granted bioRxiv a license to display the preprint in perpetuity. It is made available under aCC-BY 4.0 International license.

Exp 1 and Exp 2 correspond to two biological experiments performed with different preparations of pseudotyped viruses and one preparation of miniprotein. Error bars represent the standard error of the mean (SEM) of the technical duplicates. Fits are shown only when neutralization was detected.

bioRxiv preprint doi: https://doi.org/10.1101/2024.11.03.621760; this version posted November 4, 2024. The copyright holder for this preprint (which was not certified by peer review) is the author/funder, who has granted bioRxiv a license to display the preprint in perpetuity. It is made available under aCC-BY 4.0 International license.

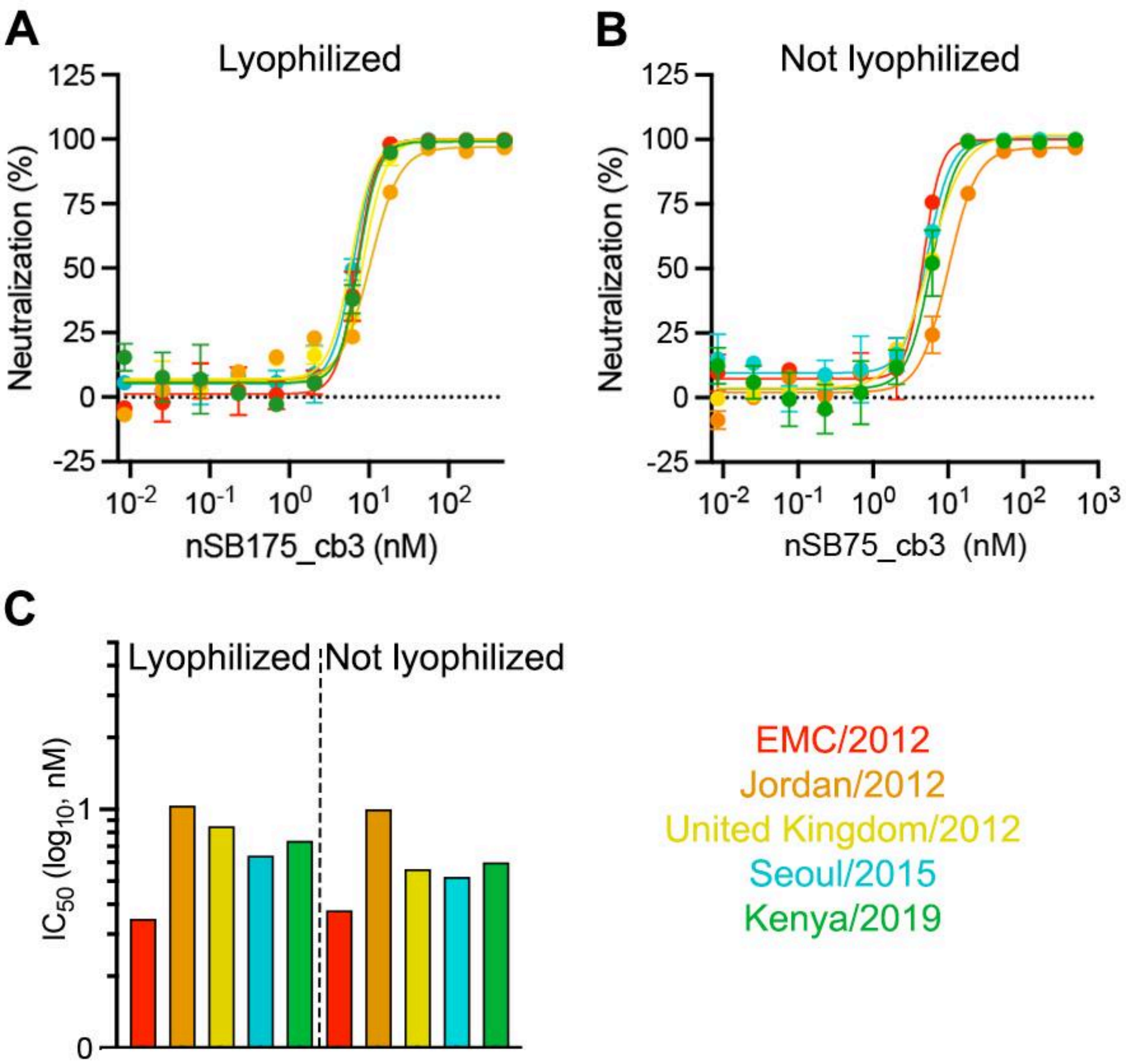

Fig S5. Retention of neutralizing activity upon miniprotein nSB175_cb3 (cb3-GSG-SB175) lyophilization. MERS-CoV EMC/2012, Jordan/2012, United Kingdom/2012, Kenya/2019 and Seoul/2015 S pseudovirus entry in the presence of various dilutions of nSB175_cb3 lyophilized and reconstituted (**A**) or not lyophilized (**B**). A single biological experiment with technical duplicates is shown. Error bars represent the standard error of the mean (SEM) of the technical duplicates. **C**. IC_{50} values, expressed in nanomolar, obtained from the experiment shown in panels A and B.

bioRxiv preprint doi: https://doi.org/10.1101/2024.11.03.621760; this version posted November 4, 2024. The copyright holder for this preprint (which was not certified by peer review) is the author/funder, who has granted bioRxiv a license to display the preprint in perpetuity. It is made available under aCC-BY 4.0 International license.

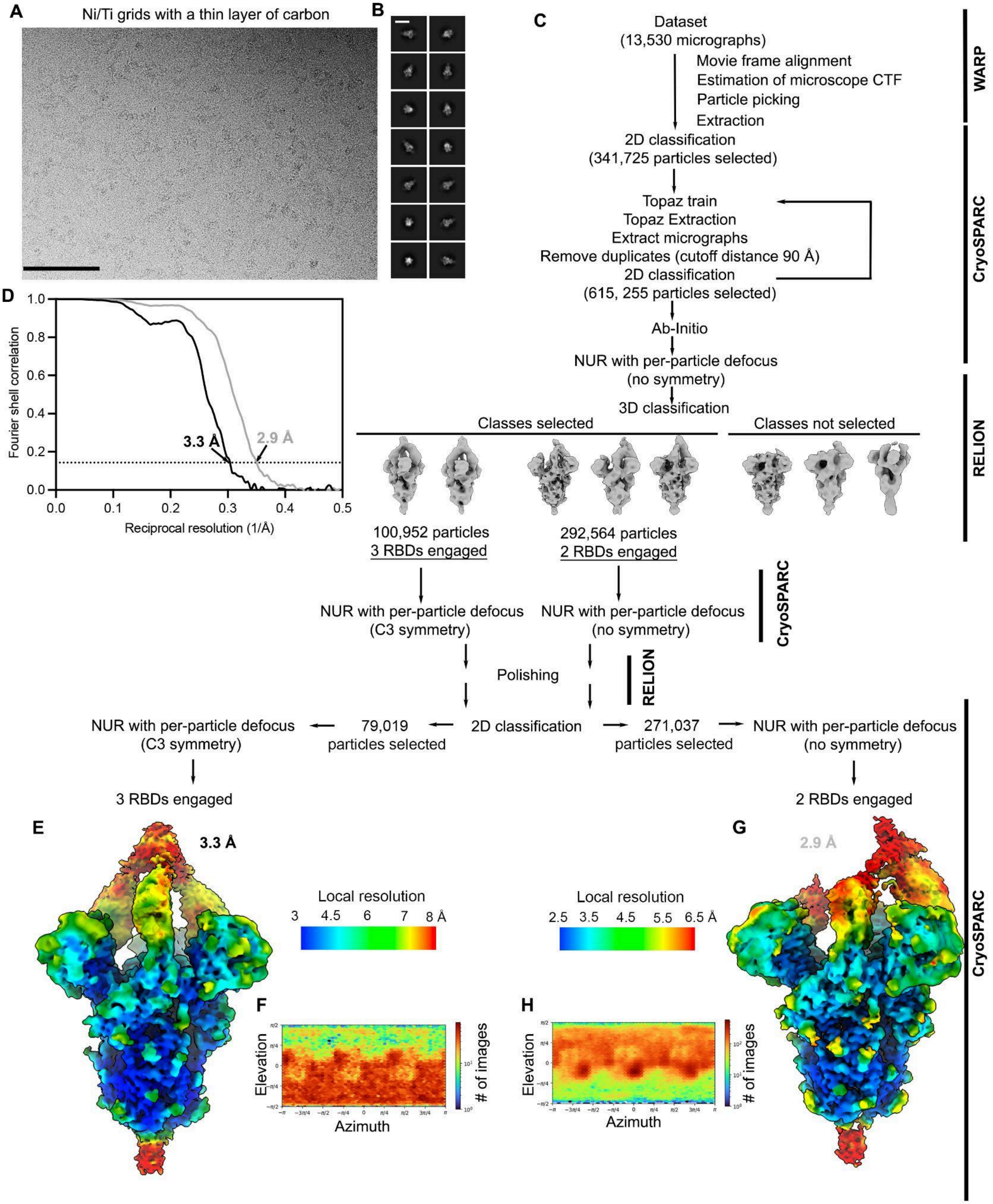

bioRxiv preprint doi: https://doi.org/10.1101/2024.11.03.621760; this version posted November 4, 2024. The copyright holder for this preprint (which was not certified by peer review) is the author/funder, who has granted bioRxiv a license to display the preprint in perpetuity. It is made available under aCC-BY 4.0 International license.

Fig S6. CryoEM data processing and validation of the structure of MERS-CoV S in prefusion conformation in complex with nSB175_cb3 (cb3-GSG-SB175). A. Representative electron micrograph. **B.** 2D class averages. Scale bar of the micrograph and the 2D class averages, 100 nm and 100 Å, respectively. **C.** Cryo-EM data processing flowchart. CTF: contrast transfer function. NUR: non uniform refinement. **D.** Gold-standard Fourier shell correlation curves for the global maps with three and two RBDs engaged are shown in black and gray, respectively. The 0.143 cutoff is indicated by a horizontal dotted black line. **E.** Unsharpened map corresponding to prefusion MERS-CoV S in complex with three nSB175_cb3 colored by local resolution. **F.** Angular distribution plot with all the particles contributing to the map in D. **G.** Unsharpened map corresponding to the prefusion MERS-CoV S in complex with two nSB175_cb3 colored by local resolution. **H.** Angular distribution plot with all the particles contributing to the map in G.

bioRxiv preprint doi: https://doi.org/10.1101/2024.11.03.621760; this version posted November 4, 2024. The copyright holder for this preprint (which was not certified by peer review) is the author/funder, who has granted bioRxiv a license to display the preprint in perpetuity. It is made available under aCC-BY 4.0 International license.

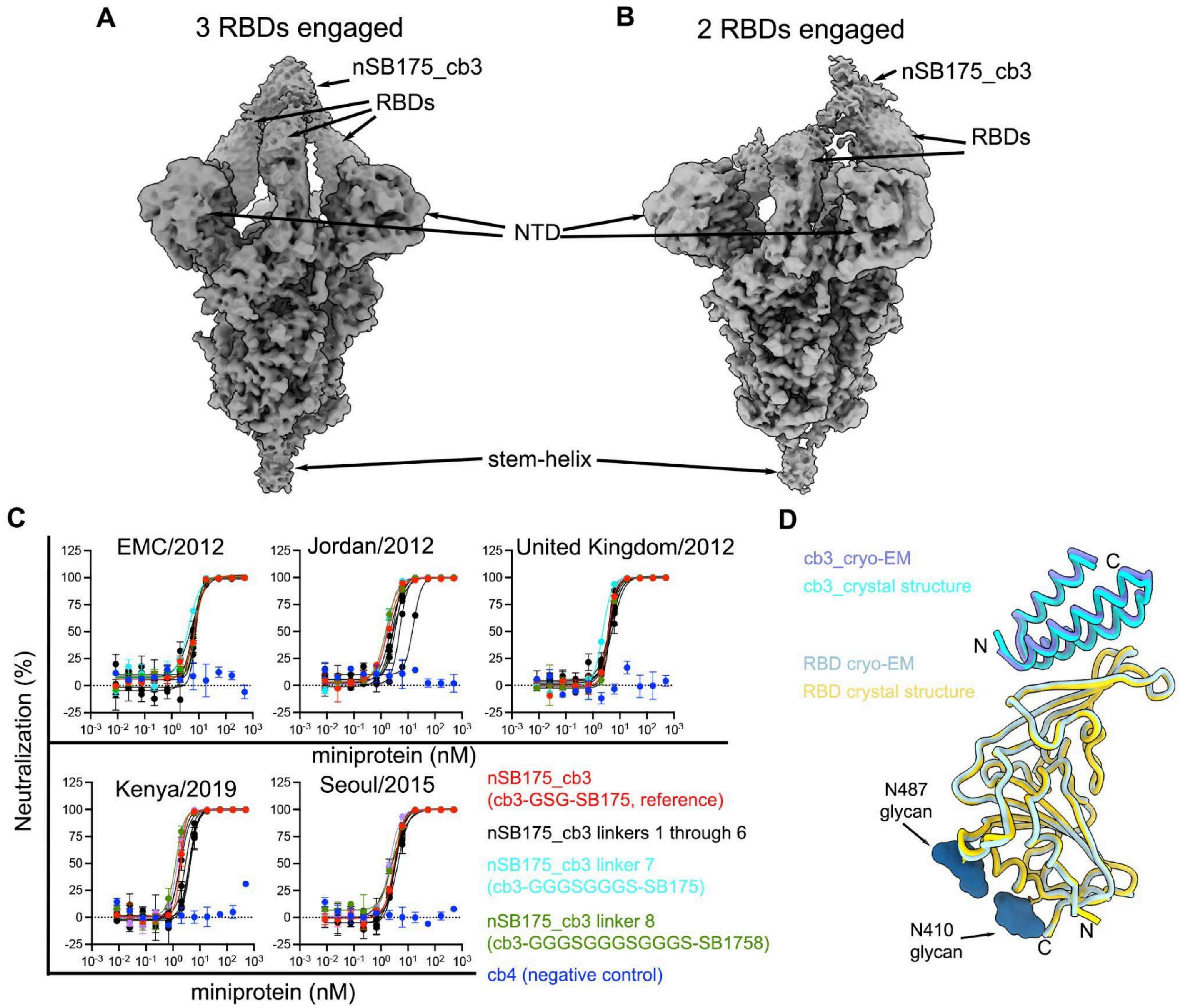

Fig S7. **Optimization of the linker length between the miniprotein binding domain cb3 and the trimerization domain SB175. A-B**. Unsharpened cryoEM maps corresponding to MERS-CoV S bound to three (**A**) or two (**B**) cb3 modules simultaneously from the nSB175_cb3 homotrimeric miniprotein. **C.** MERS-CoV EMC/2012, Jordan/2012, United Kingdom/2012, Kenya/2019 and Seoul/2015 S VSV pseudovirus entry into cells in the presence of various dilutions of nSB175_cb3 with different linkers sizes between cb3 and the trimerization domain. Miniprotein cb4 was used as a negative control. A single biological experiment with two technical replicates is shown. Error bars represent the standard error of the mean (SEM) of the technical duplicates. **D.** Structural overlay between the cryo-EM structure of the MERS-CoV S RBD in complex with nSB175_cb3 linker 7 and the X-ray structure of the MERS-CoV S RBD in complex with a single cb3.

bioRxiv preprint doi: https://doi.org/10.1101/2024.11.03.621760; this version posted November 4, 2024. The copyright holder for this preprint (which was not certified by peer review) is the author/funder, who has granted bioRxiv a license to display the preprint in perpetuity. It is made available under aCC-BY 4.0 International license.

bioRxiv preprint doi: https://doi.org/10.1101/2024.11.03.621760; this version posted November 4, 2024. The copyright holder for this preprint (which was not certified by peer review) is the author/funder, who has granted bioRxiv a license to display the preprint in perpetuity. It is made available under aCC-BY 4.0 International license.

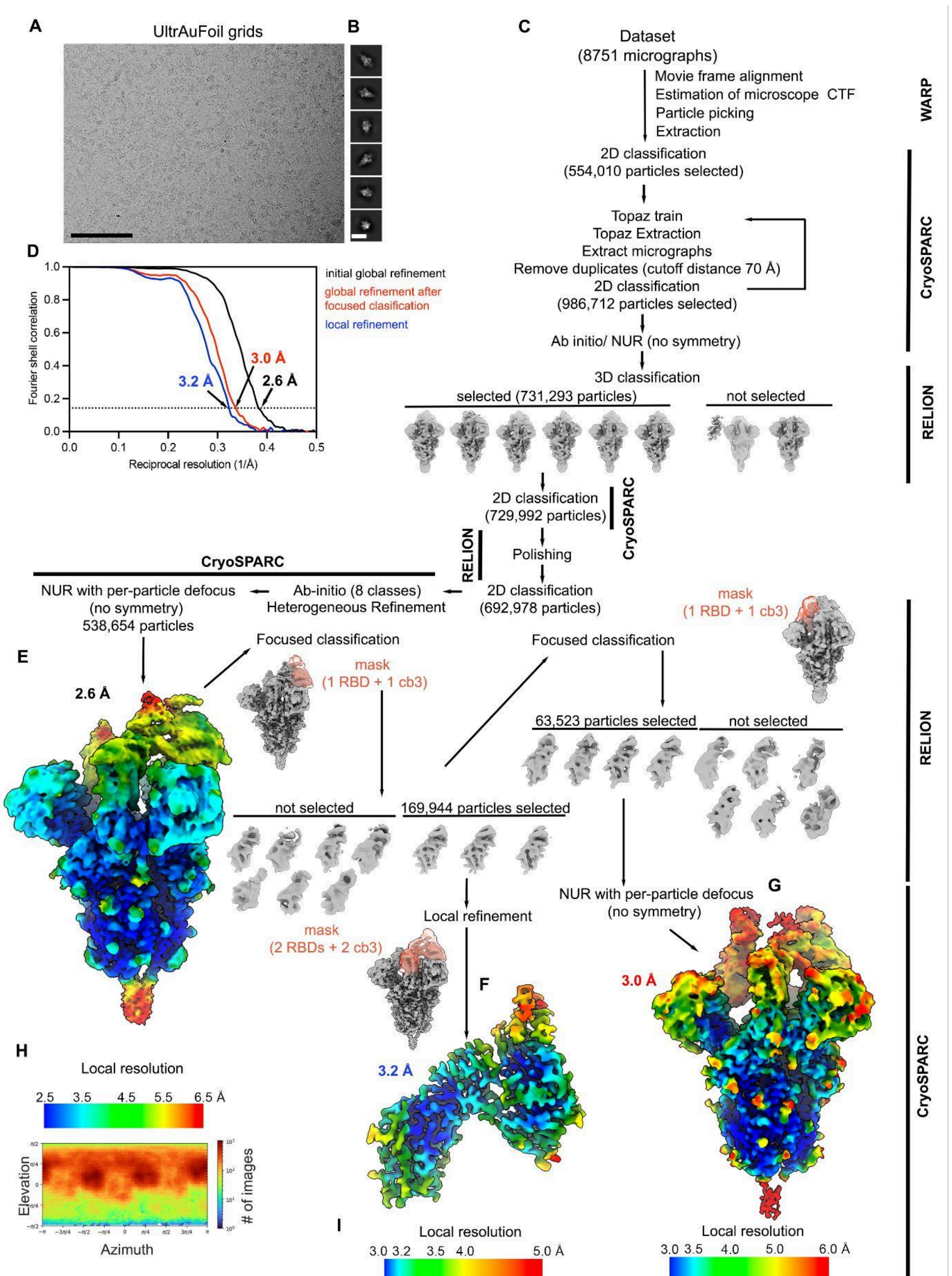

bioRxiv preprint doi: https://doi.org/10.1101/2024.11.03.621760; this version posted November 4, 2024. The copyright holder for this preprint (which was not certified by peer review) is the author/funder, who has granted bioRxiv a license to display the preprint in perpetuity. It is made available under aCC-BY 4.0 International license.

Fig S8. CryoEM data processing and validation of the structure of prefusion MERS-CoV S in complex with nSB175_cb3 linker 7 (cb3_GGGSGGGS_SB175). A. Representative electron micrograph. **B.** 2D class averages. Scale bar of the micrograph and the 2D class averages, 100 nm and 100 Å, respectively. **C.** Cryo-EM data processing flowchart. CTF: contrast transfer function. NUR: non uniform refinement. **D**. Gold-standard Fourier shell correlation curves for the global maps (black and red) and locally refined map (blue). The 0.143 cutoff is indicated by a horizontal dotted black line. **E.** Unsharpened map corresponding to the 3D reconstruction of MERS-CoV S (in prefusion conformation) in complex with nSB175_cb3 linker 7 colored by resolution. **F.** Local refined sharpened map corresponding to two MERS-CoV S RBDs engaging two cb3 from the nSB175_cb3 linker 7 miniprotein colored by resolution. **G.** Global unsharpened map for the MERS-CoV-S in complex with nSB175_cb3 linker 7 miniprotein obtained after focused classification and colored by resolution. **H, I.** Angular distribution plots corresponding to the maps shown directly above.

bioRxiv preprint doi: https://doi.org/10.1101/2024.11.03.621760; this version posted November 4, 2024. The copyright holder for this preprint (which was not certified by peer review) is the author/funder, who has granted bioRxiv a license to display the preprint in perpetuity. It is made available under aCC-BY 4.0 International license.

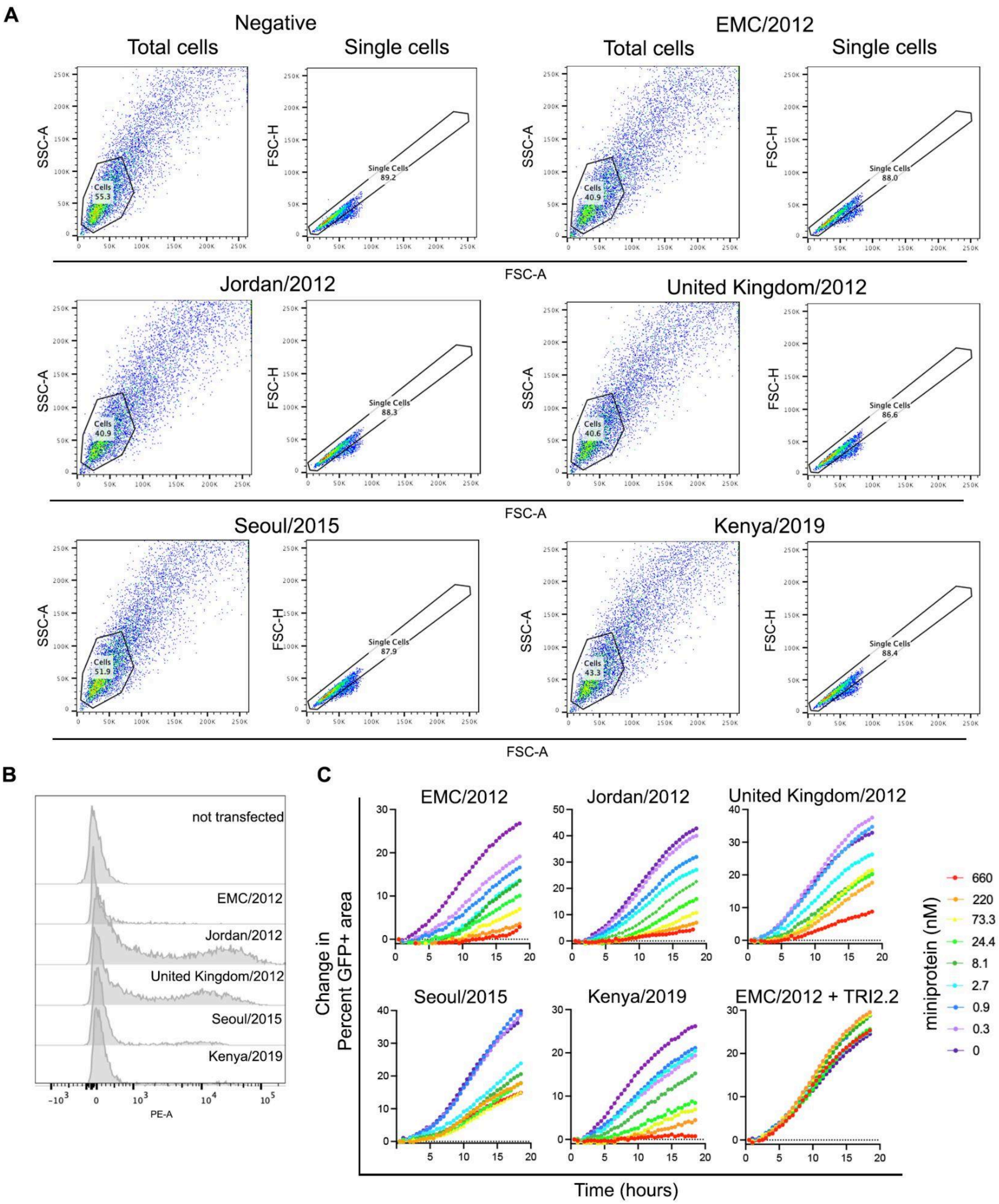

bioRxiv preprint doi: https://doi.org/10.1101/2024.11.03.621760; this version posted November 4, 2024. The copyright holder for this preprint (which was not certified by peer review) is the author/funder, who has granted bioRxiv a license to display the preprint in perpetuity. It is made available under aCC-BY 4.0 International license.

Fig S9. nSB175_cb3 (cb3-GSG-SB175) inhibits MERS-CoV S-mediated membrane fusion. A. Gating strategy for flow cytometry analysis of cell-surface expression of MERS-CoV S proteins from EMC/2012, Jordan/2012, United Kingdom/2012, Kenya/2019 and Seoul/2015 strains expressed at the surface of BHK-21-GFP$_{1-10}$ cells. Representative gating to exclude cell debris and dead cells (SSC-A/FSC-A, total cells) and to select single cells (SSC-H/FSC-A, single cells) are shown. **B.** Quantification by flow cytometry of the different MERS-CoV S surface expressed on BHK-21-GFP$_{1-10}$ using the stem helix antibody B6*(48)*. The y-axis is present as a modal scale scaled to maximum singleton events for that plot. **C**. Kinetics of cell-cell fusion (expressed as a change in percent of GFP^{+} area) promoted by the different MERS-CoV S glycoproteins over an 18 h time course experiment using a split GFP system and in the presence or absence of different concentrations (expressed in nM) of cb3-GSG-SB175 or TRI2.2. Data represent one experiment out of two biological replicates.

bioRxiv preprint doi: https://doi.org/10.1101/2024.11.03.621760; this version posted November 4, 2024. The copyright holder for this preprint (which was not certified by peer review) is the author/funder, who has granted bioRxiv a license to display the preprint in perpetuity. It is made available under aCC-BY 4.0 International license.

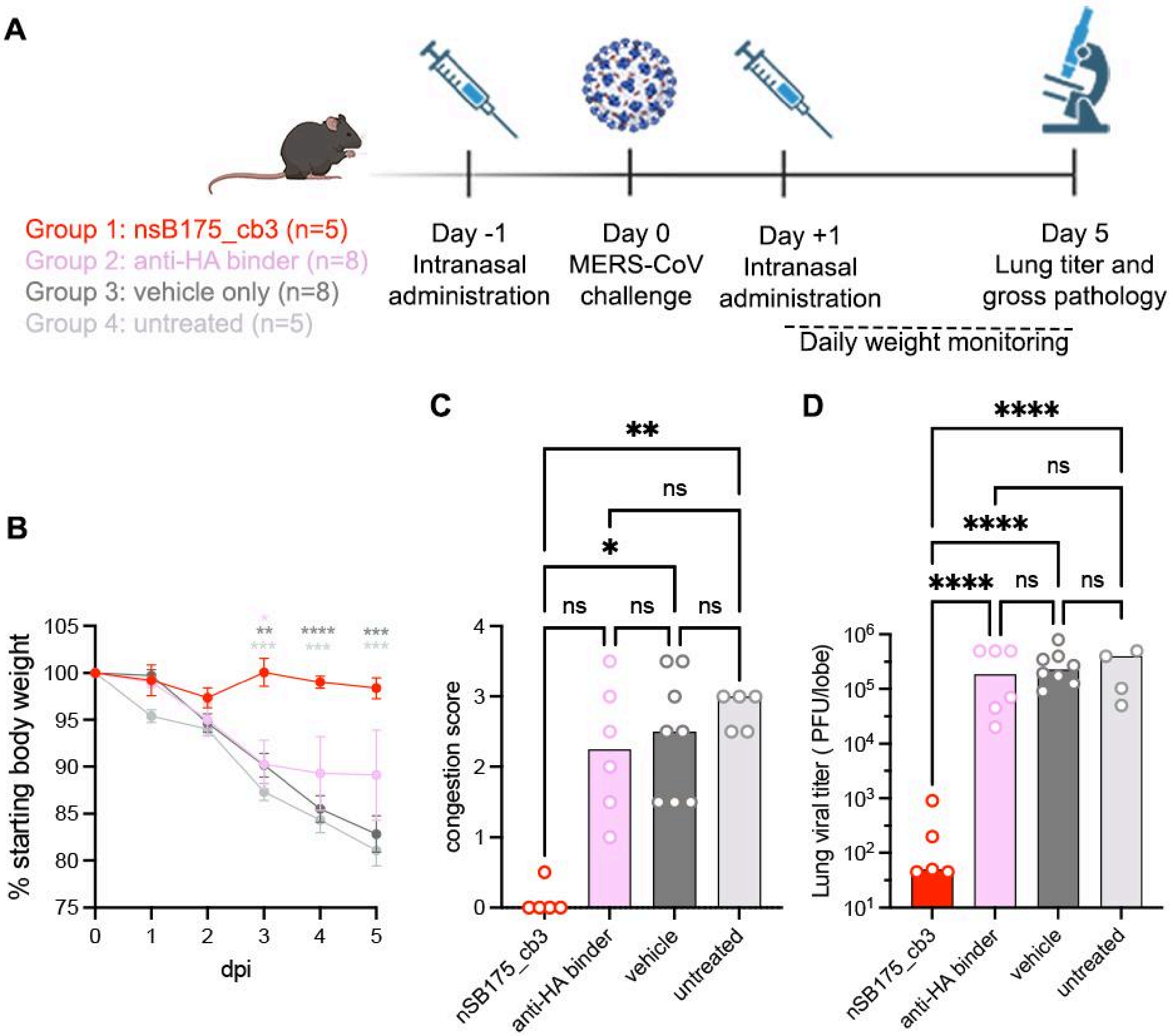

Fig S10. Pre- and post-administration of nSB175_cb3 (cb3-GSG-SB175) protects mice from MERS-CoV challenge. **A.** Trimeric fusion nSB175_cb3 (cb3-GSG-SB175), influenza hemagglutinin miniproteins (anti-HA), or vehicle alone were administered to mice C57BL/6 J 288/330 at day -1 and day +1. An untreated group was also included. Mice were challenged with MERS-CoV-m34c5 at day 0 and monitored for 5 days for body weight (**B**) or analyzed at day 5 for congestion score (**C**) and viral titer in lungs (**D**). Group comparisons for body weight and viral titers were assessed with the two-way ANOVA: Tukey's test. For the congestion score, comparisons among groups were assessed with the Kruskal-Wallis test; ns, not significant; $^*P < 0.05$, $^{**}P < 0.01$, $^{****}P < 0.0001$.
